# Variations in Soil Blue Carbon Sequestration between Natural Mangrove Metapopulations and a Mixed Mangrove Plantation: A Case Study from the World’s Largest Contiguous Mangrove Forest

**DOI:** 10.3390/life13020271

**Published:** 2023-01-18

**Authors:** Abhiroop Chowdhury, Aliya Naz, Subodh Kumar Maiti

**Affiliations:** 1 Jindal School of Environment and Sustainability, O.P. Jindal Global University, Sonipat 131001, Haryana, India; 2 Ecological Restoration Laboratory, Department of Environmental Science & Engineering, Indian Institute of Technology (ISM), Dhanbad 826004, Jharkhand, India; 3 Jindal School of Liberal Arts and Humanities, O.P. Jindal Global University, Sonipat 131001, Haryana, India

**Keywords:** mangrove, restoration, blue carbon, degraded mudflat, plantation, ecological function, ecological service, island biogeography

## Abstract

Sundarban is the world’s largest mangrove wetland. This study, conducted in 2016, to compare blue carbon sequestration with different natural metapopulations and a four-year-old *Avicennia marina* (30% area)-*Rhizophora mucronata* (70% area)-mixed mangrove plantation under anthropoganic stress. The aims of the study is to find out the variations in soil ecological function indicators (pH, electrical conductivity, bulk density, soil texture, available nitrogn, phosphorus and soil organic carbon) and key ecological service indicator (soil blue carbon pool) between sites. Simpson’s Index of dominance, diversity and Shannon-Weiner Index revealed that all the sites are under ecological stress, with the *Suaeda maritima*-dominated mudflat having the least biodiversity. It is also revealed that pH and electrical conductivity were highest in *Suaeda maritima* and *Phoenix padulosa*-dominated metapopulations, whereas organic carbon was the highest under the mangrove plantation and *Avicennia marina*-dominated site. Available nitrogen was recorded highest in the community with the *Sonneretia* sp.-*Avicennia marina* association. The mixed mangrove plantation had the highest blue carbon pool. The species diversity was not found to be related with the distance from the nearby conserved mangrove forest, contrary to the island biogeography theory. This study concludes with a recommendation of mixed mangrove plantations to restore the degraded saline mudflats along the human settlements across the globe.

## 1. Introduction

Mangroves are specially adapted plant groups that have a physiological, morphological, or anatomical adaptation to survive and proliferate in saline tide washed soil and mudflats. Mangroves are distributed across the globe in the tropical and subtropical coastlines, deltas, estuaries, and islands. Indonesia has the largest area under the mangrove cover scattered across the tropical islands, but the ‘Sundarban mangrove wetland’ shared by Bangladesh (60%) and India (40%), is the world’s largest contiguous mangrove forest. This region is also the world’s largest delta with dense population, facing intense anthropocentric developmental pressures.

Existing in the ecotone of terrestrial and marine ecosystems, mangroves are crucial for the survival and healthy functioning of both. Research has already established a multitude of ecological services rendered by these plant communities, namely, Ecosystem-based Disaster Risk Reduction (Eco-DRR), breeding ground of marine fauna, pollutant sink, economic service in terms of forest products, consolidation of soil, and resilience to tidal erosion [1,2,3,4,5,6]. Foremost amongst these services is their ability to sequester an enormous amount of carbon in the soil. This sequestered soil carbon in the saline, edaphic environment of mangrove and sea grasses is referred as ‘blue carbon’. Mangrove and sea grass constitutes the blue carbon sink, which is larger than most of the terrestrial ecosystems, such as boreal forests and tropical forest types [7]. Blue carbon has been recognized as a solution to the global problem of climate change. Sundarban ecosystem is a repository of the sediment organic carbon content, varying between 0.92 to 3.29%, resulting in an organic carbon stock of 59.14 Tg within 90 cm of the intertidal sediment [6].

With all the conservation efforts and global focus on conserving the mangrove ecosystem, the footprint of human development has altered the land use and mangrove cover across the globe [8]. By then, 54 out of 102 islands at the Indian part of Sundarbans was under human habitation and mangrove forests had been wiped out to facilitate developmental activities such as agriculture and aquaculture. The rest of the islands were conserved and is the last remaining frontier for enigmatic mangrove forest dwelling Royal Bengal Tigers. However, blue carbon stocks are reducing across the Sundarban mangrove ecosystem [6,9]. Mangroves in an average can sequester of 1023 Mg C per hectare. Studies demonstrate that Sundarban’s mangrove ecosystem can sequester approximately 4.71–6.54 Mg C ha^−1^ year^−1^ C [6,9]. However, land conversions and climate change impacts are destabilizing both ecological functions and services at Indian Sundarbans. Research by Bera et al. revealed an approximate 6,313,944 mg/6.31 Tg loss of C at Sundarbans between 1975 and 2020 [9].

The recent decade has observed a shift in international perception towards climate change and global warming. The frequency and number of tropical disturbances had also increased over the years due to the impact of climate change, periodically inundating human-colonized islands of Indian Sundarbans. Restoration initiatives have also increased in these islands to protect the mudflats and embankments from erosion during natural disasters [5]. Blue carbon sequestration is the key tool in the hand of climate change ecologists against the fight with climate change. Restoration initiatives are known to improve the blue carbon pool [3].

This article focuses on a research question: ‘Does multispecies mangrove plantation help in improving blue carbon sequestration in comparison to natural mangrove patches under anthropogenic pressure?’. The rationale of the study is to compare the soil carbon pool between different mangrove metapopulations (with varied community composition) to a mixed mangrove plantation.

To answer this question, our lab group investigated the biodiversity and blue carbon pool along with associated soil ecological function indicators between five sites with different mangrove community composition, of which one site is a 4-year-old mixed species plantation.

## 2. Materials and Methods

### 2.1. Study Site

**Figure 1:**
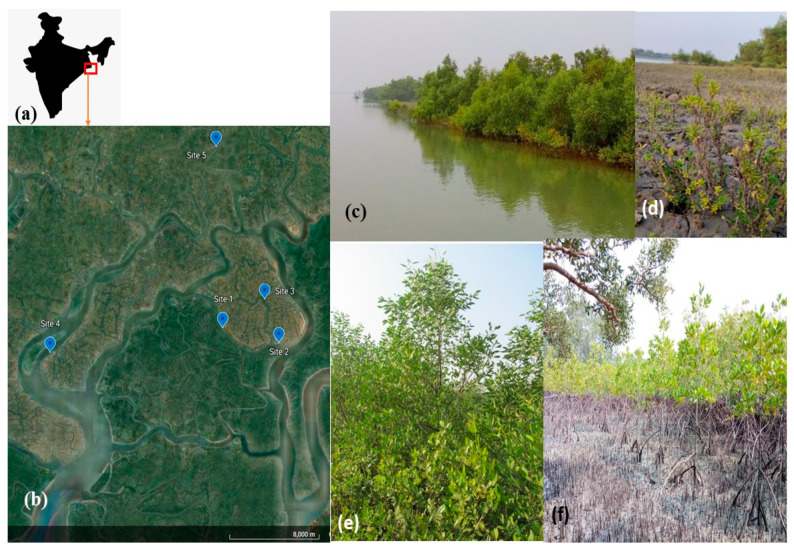
The study site. (**a**) From map of India, the study area has been zoomed out to represent all the sampling sites in a google earth image. (**b**) Imagery of the study site locating the sampling metapopulations. (**c**) Site-2 (S2) dominated by *A. marina* strands. (**d**) Site-5 (S5) metapopulation dominated by halophyte-*S. maritima*. (**e**) Site-3 (S3) dominated by *A. marina*-*S. caseolaris* association and (**f**) Plantation site with establishment of *R. mucronata* stands.

The study area has been selected at different parts of Indian Sundarbans with different forest/plantation, as natural mangrove community sites. Descriptions and locations of the sites have been elucidated in Table 1 and Figure 1.

### 2.2. Plantation, Monitoring and Assessment Strategy

**Figure 2:**
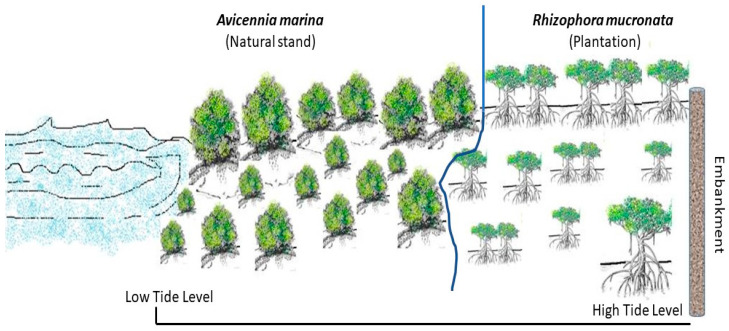
The plantation site (S1) species composition. The Low Tide Level (LTL) side represented by water body has the established natural stand of *A. marina* (approximately 30% area cover) followed by the plantation of *R. mucronata* (approximately 70% area cover).

Site-1: *Rhizophora mucronata* have been planted through the drain and trench method to maximize the survival of the nursery grown saplings [3]. Before the *R. mucronata* plantation site, there was a natural cover of *Avicennia marina* plants. The location of the plant layers with respect to High Tide Level (HTL) and Low Tide Level (LTL) line has been depicted in Figure 2.

Site-2–5: Natural degraded mudflats near to the plantation site with about 70% mudflat covered with a particular mangrove or halophyte species. Samplings and biodiversity survey have been conducted on 2016 (Post-monsoon). As this site is devoid of human intervention or plantation program, the community ecology of the mangrove metapopulation was liable to change under natural influences. Hence, there has been a need for a yearly biodiversity assessment in the area. Five quadrat plots (10 m × 10 m) were randomly set in the site each year and the average individual of the recorded species was used to assess the biodiversity.

The species diversity of the sites was assessed through Relative density and Frequency as per previous research on community ecology at the Indian Sundarbans [10].

Relative density is an estimate of the numerical strength of a species in relation to all the individuals of all the species, defined as;

$$\mathrm{Relative}\;\mathrm{Density}\;\left(\mathrm{RD}\right)=\frac{\mathrm{Number}\;\mathrm{of}\;\mathrm{individuals}\;\mathrm{of}\;a\;\mathrm{species}}{\mathrm{Number}\;\mathrm{of}\;\mathrm{individuals}\;\mathrm{of}\;\mathrm{all}\;\mathrm{the}\;\mathrm{species}}\times 100$$

The distribution or dispersion of a individual species is generally estimated as percentage occurrence and is defined as;

$$\mathrm{Frequency}\;\left(\%\right)=\frac{\mathrm{Number}\;\mathrm{of}\;\mathrm{quadrats}\;\mathrm{the}\;\mathrm{species}\;\mathrm{occured}}{\mathrm{Total}\;\mathrm{number}\;\mathrm{of}\;\mathrm{quadrat}\;\mathrm{studied}}\times 100$$

Both the estimators give an idea of the status of a particular species in relation to the whole community, which is used in this study to understand the changes in distribution of the plants.

### 2.3. Soil Sampling and Analyses

Soil sampling was conducted in the sites during 2016. An acid washed trowel was used to scoop out the soil from upper surface of the mudflat (0–30 cm). The sample was collected in three replicate subsamples for each five sites, which were further mixed and homogenized to prepare a composite soil sample. The sites were selected, keeping in mind the similarity of anthropogenic stresses at all sites (to negate the biasness of the data) and to cover maximum types of mangrove community composition found at human-inhabited parts of the Indian Sundarbans. In the plantation sites, soil samples were collected from the four corners of ten 5 m × 5 m quadrat, of which three were from areas with *A. marina* dominance and seven from areas with *R. mucronata* dominance, and all the soil samples are mixed. Approximately 30% of the area was under the cover of *A. marina*, while the rest of the 70% was under the *R. mucronata* plantation, though there was intermixing at most of the sampled spaces. The collected soil samples were placed in plastic bags and labeled before being brought to the laboratory.

Soil organic carbon (SOC) was determined through the wet digestion method and reported as a percentage [11,12]. While preparing the reagents for analysis of SOC, 5 g Ag_2_SO_4_/L of H_2_SO_4_ is added before the use of H_2_SO_4_ to minimize the interference of Cl^-^ in the saline mangrove soil [3]. Bulk density was calculated through the standard method [12].

Soil pH was measured by a multiparameter pH probe (HI-2020, Hanna Instruments, India) by making a suspension with deionized water (1:2.5, *w*/*v*) and after allowing it to settle for one hour [12]. Plant-available nitrogen (N) was estimated after digesting the samples with 0.32% KMnO_4_ solution followed by titration with 0.02 N H_2_SO_4_ using the Kjeldahl distillation unit (KJELODIST-EAS VA, Pelican Equipment Inc., Chennai, India) [13]. Plant available Phosphorus was determined by the Olsen method [14].

Carbon pool (0–10 cm) was calculated using SOC concentration, bulk density, and particular soil depth [15], as follows:

Carbon pool (Mg C ha^−1^) = SOC × BD × T

where SOC = Soil Organic carbon (%); BD = bulk density (g cm^− 3^), and T = soil thickness (cm).

### 2.4. Assessing the Health of the Ecosystem

‘Community ecology’ has two estimators that indirectly shed light onto the overall health of the ecosystem. The first one is a ‘Dominance’ estimator that focuses on overall dominance of one or group of species representing α-diversity of the ecosystem. The reciprocal of the dominance estimator is the ‘Diversity’ value. However, these estimators are generally biased to the most dominant species in an ecosystem. Hence, information-statistic indices are used to quantify the ‘entropy’ or ‘randomness’ in a community, which shed light onto the overall health of an ecosystem.

For understanding dominance, ‘Simpson’s Index of Dominance (D)’ is used, which is [16,17],

$$D=\Sigma \frac{n\left(n-1\right)}{N\left(N-1\right)}$$

Simpson’s Index of Diversity (L) is [16,17],

L=1−D

Shannon Diversity Index (H) shed light into the overall diversity and health of the ecosystem, depicted as [18],

H=−Σ piLn pi

In the equations, D = Simpson’s Index of dominance, L = Simpson’s Index of Diversity, H’ = Shannon’s Index, n = Number of individuals of each species, N = Total number of individuals of all the species, and pi = Total number of individuals in each species/Total number of individuals in all the species [18].

### 2.5. Statistical Tests

Statistical treatment was applied in Microsoft Excel 2007 and SPSS 16 (SPSS Inc. Chicago, IL, USA). The regression analysis was used to understand the trends in temporal changes in salinity regime and biodiversity. The standard error is used to understand the reliability of the data. The variance of mean (n = 3) has been evaluated by the Analysis of Variance (one-way ANOVA), followed by Duncan’s post hoc test after testing the dataset for the application of these statistics using the Shapiro–Wilk’s and Levene’s test.

## 3. Results

### 3.1. Biodiversity and Ecological Health Assessment

Blue carbon has been referred to as one of the key ecosystem services of mangroves, and scientists across the globe has been trying find out the relationship between the sequestration and drivers that influence the rate of C-trapping in the soil [19]. The biodiversity assessment in the sites revealed an array of species composition, where their relative density and frequency has been depicted in Table 2.

The health of the ecosystems in all the five sites has been elucidated by Simpson’s index of Dominance and Diversity, and the information-statistic index-Shannon Weiner Index (Table 3).

Similar studies use these quantitative ecological estimators to shed light onto the health of the ecosystem [10,20,21].

### 3.2. Soil Parameters

Soil parameters such as soil salinity, pH, bulk density (BD), soil organic carbon (SOC), available nitrogen (N), available phosphorous (P) and soil texture have been analyzed to quantify ecological functions. The results depicted in Table 4 indicate that these parameters are statistically and significantly varying across the five sites (S1–S5).

## 4. Discussion

### 4.1. Biodiversity and Ecological Health

It is evident from Table 2 that the Plantation location (Site 1 or S1) has a 24 RD value for *A. marina*, while it has an RD of 60 for *R. mucronata* (planted species). This species association is due to the mixed plantation model with naturally existing *A. marina* trees in the Low Tide Level, to reduce the wave impact during the tides. This resulted in luxuriant growth of *R. mucronata*. The mangrove associate *A. ilicifolius* was only nominally present in the site. 

Site-2 (S2) has an 80 RD value for *A. marina*, indicating its dominance in the natural metapopulation. The rest of the species are rarely present in the site. Site-3 has a 48 RD for *A. marina,* while *S. caseolaris* is recorded to be at 16. *A. ilicifolius* have a RD of 24, but it is a small thorny herb and a mangrove associate (semelparous species), while both *A. marina* and *S. caseolaris* are trees with an iteroparous life cycle pattern. The Semelparous species shows a life strategy where the plants (generally herbs) grow for a short duration (seasonal or annual), and invests maximum energetics in the reproductive cycle and propagation. The short life cycle makes it difficult for semelparous species to impart any changes in the microenvironment where they grow. Iteroparous species can live longer and obtain reproductive maturity at a latter part of life but can modify the microenvironment, making it suitable for growth. Mostly tree species follow an iteroparous life cycle.

Site-3 (S3) has an association of two iteroparous tree species: *A. marina* (RD = 55) and *S. caseolaris* (RD = 21). Though the semelparous mangrove associate and grass *P. coarctata* have a RD value of around 13, it minimally impacts the microenvironment of the habitat.

Site-4 (S4) has a dominance of the edaphic sub-climax species and mangrove palm and shrub, *P. paludosa* (RD = 63). The rest of the species have a sparce distribution. It does indicate a greater erosional impact in the study site, as focused on previous studies [22]. These shrublands provide little protection against the tidal current or wave action during storm surges, making the shorelines vulnerable to erosional dynamics, unlike iteroparous mangrove trees such as *A. marina*, *R. mucronata*, *C. tagal* or *S. caseolaris*, which impart ecosystem services such as the consolidation of soil, barrier against natural disaster, and protection from erosion [5].

Site-5 (S5) has a a dominance of only a salt tolerant ruderal semelparous herb-*S. maritima* (RD = 69). The rest of the species has been sparsely distributed in the study site. This species has been demonstrated to be highly adapted to soil salinity and oxygenation stress [23]. They are generally observed in the sites under salinity stress with low biodiversity. It creates a mat in the soil, making it difficult for propagules of other species to lodge and grow in the sites. Being a semelparous ruderal, it rarely influences the micro-environment of the study site.

The theory of island biogeography suggests that the distance from the nearest large population pool determines the distribution of species [24]. However, in the case of Sundarbans, this theory may not be justified. In the current study, it has been observed that the distance from the nearby conserved larger metapopulation does not determine the species distribution of mangroves (Table 1).

This spatial study indicates that the distance from the nearest conserved mangrove population and observed species number at the sites indicates that geological forcing of environmental parameters may be more important for the species distribution of mangroves, contrary to the island biogeography theory. Though data from only five sites do not conclusively disprove the island biogeography theory at Indian Sundarbans and that geological, climate change, ecological forcing have been more prominent drivers for shaping the mangrove community ecology, but it does substantiate further investigation on understanding the ecosystem of Sundarbans through the lens of ‘Island biogeography’ theory.

The plantation site (Site-1) shows a higher diversity and lower dominance value, indicating a restoration of the ecosystem with different species colonizing the sites apart from the planted species-*R. mucronata*. The Shannon-Weiner Index shows a higher comparative value compared to other natural sites except Site-3. Amongst all the sites, Site-3 with a natural assemblage of *A. marina* and *S. caseolaris* had the robust ecosystem health and lesser randomness or ‘entropy’ in the ecosystem. The Diversity value is also the highest amongst other sites, which is reciprocal to the dominance. The sites (4 and 5) under erosional or salt stress and with only dominant semelparous herbs, had the lowest diversity value and highest randomness or entropy amongst all the α-diversity habitats. This concludes that both the sites are under stress, with a local extinction of most of the key mangrove species found in the Sundarban mangrove community ecology such as *B. sexangula*, *C. tagal*, *R. mucronata*, *S. caseolaris*. The Shannon-Weiner Index value ideally ranges between 1.5 to 3.5. This value of Shannon-Weiner for all the sites is below, 1.5, which indicates increased anthropogenic stress on all the sites. The selection of study site is based on the mudflats near to human habitation; hence, anthropogenic stresses were essential drivers in shaping the community ecology of all the sites.

### 4.2. Comparing the Ecosystem Function and Services

Table 4 indicates the major indicators of ecosystem services such as pH, soil salinity, OC, BD, N, P and soil texture. The ecosystem function and services can be quantified through these soil indicators [25,26]. S1 have the highest proportion of clay, while S3 have more silt and S4 have the highest sand proportion amongst all the sites. The pH of all the sites varied significantly and was recorded within the alkaline range. The pH of the soil in S4 and S5 is highly alkaline, and the same sites also have the high soil salinity in comparison with the other sites. The pH of hypersaline mangrove wetlands always tends to be alkaline due to the presence of basic ions of Na, K, Ca and Mg. The high salinity and pH in S4, S5 indicates the formation of ‘saline blanks’ on the surface of the soil. This occurs when a particular soil has been devoid of biodiversity cover, and that results in the transport and deposition of salt from lower depths of soil through capillary action due to evaporation. Both S4 and S5 has been observed to have saline blanks; although, in the case of S5, around 20% of the surveyed land is impacted due to the saline blank formation. High salinity and pH hinder the growth and colonization of natural mangrove species; hence, both the sites have a dominance of only salt tolerant or stress tolerant semelparous species, instead of a healthy mangrove community composition. This has been evident from the Shannon-Weiner Index and Simpson’s Index of Diversity.

Soil organic carbon is highest in the S1, due to the association between two key mangrove species. The ecosystem has been restored, which is evident from the mixed species composition and colonization trend of non-planted mangroves and halophytes. S2, which have a dominance of the salt tolerant mangrove, *A. marina*, also recorded a high SOC compared to other sites. *A. marina* grows fast and has been known to tolerate high salinity stress, and is hence ideal for proliferation at Sundarban mudflats [10,27]. The other ecosystem function parameters such as N and P have also been recorded higher in S1, S2 and S3 compared to the other. All of these sites have a dominance of species, which are iteroparous mangroves; hence, the leaf litter is accumulated and transported to soil daily. All mangroves are evergreen plants that shed leaves throughout the year, unlike deciduous plants. The litter constitutes for increased nutrient-N,P in the soil. When compared between all five metapopulations considered in this study, the highest N is recorded in S3 because of the association between two mangrove tree species—*A. marina* and *S. caseolaris*. Previous studies have demonstrated that the *S. caseolaris* leaf has an N content that influences the nutrient dynamics of the soil where it grows [28]. The association of the two species and colonization of the other mangrove species indicates an approximately better ecological health, but a Shannon-Weiner value less than 1.5 for all the sites does indicate a stressed ecosystem that is in need of conservation interventions.

**Figure 3:**
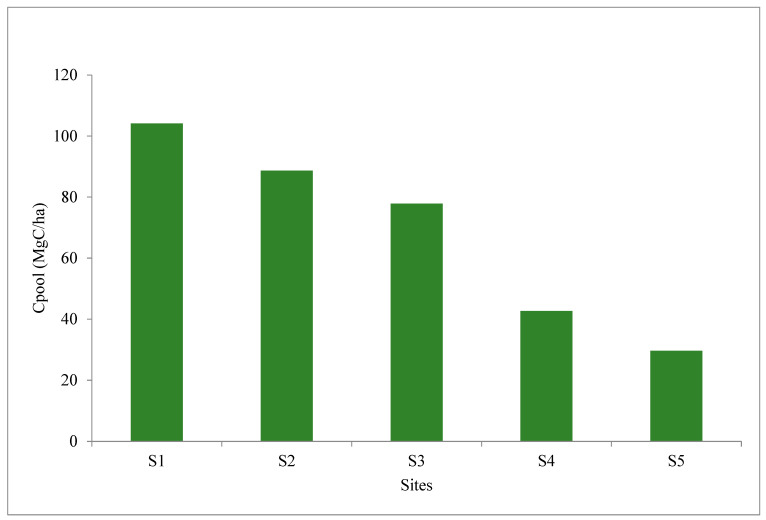
A comparison between the ‘Blue carbon’ pool between the sites.

The blue carbon pool was found to be highest in the plantation site (S1), followed by S2 with more than 10–15-year-old *A. marina* strands (Figure 3).

Multispecies strands and restoration initiatives through the multispecies mangrove does improve upon the blue carbon sequestration in soil compared to degraded mudflats under the dominance of stress tolerant semelparous halophytes or mangrove associates (S4, S5).

Similar restoration initiatives have been observed throughout the mangrove habitats across the globe. The average soil organic carbon stock and N for the top 1 m soils has been observed to be 263 ± 14 Mg C ha^−1^ and 11.8 ± 0.4 Mg N ha^−1^ at eastern Brazilian mangroves [29]. A restoration initiative at Xiamen, China, revealed that the 10-year-old *Sonneratia apetala* and *Kandelia obovata* plantations have a C-sequestration rate of 3.32 ± 0.62 kg C m^−2^ yr^−1^ [30]. An investigation of soil carbon sequestration and the carbon storage of mangroves and *Spartina* spp. on Maoyan and Ximen islands indicated that the *Spartina* spp. can store 12.7–31.4% more carbon than the naked mudflat [31]. A conservation project at the Gulf of California, Mexico protected 16,058 ha of mangroves, equivalent to 2.84 million Mg CO_2_ emission reduction within a period of 100 years [32]. A total of 22% of the conserved mangrove forests of Indonesia resulted in 0.82–1.09 PgC hectare^−1^ of carbon storage [33]. A *Sonneratia caseolaris* and *S. apetala* mixed species plantation in southern China have recorded an increased C-stock in the sediment compared to monocultures, as is also evident from this study [34]. Hence, mangrove restoration is one of the most effective methods to combat the vagaries of global warming by improving the C-sequestration.

## 5. Conclusions

This research concluded that plantations with multispecies iteroparous mangroves improve the ecological functions and services of the degraded mudflats under anthropogenic stress. The plantation sites have a better blue carbon pool compared to others. *P. paludosa* and *S. maritima* have been indicative of a degraded, eroded, or stressed habitat, and such mudflats can be a potential site for the multispecies mangrove eco-restoration initiatives. The species diversity does not follow the island biogeography theory, which indicats a possible geological or environmental driver behind the mangrove species distribution, substantiating the need for future research. Ecological indicators need to be incorporated in the decision making along with a regular in situ mangrove biodiversity assessment to grasp the level of degradation in the mudflats across the human-inhabited parts of the Indian Sundarbans. This study also recommends further research at Sundarbans as well as other mangrove multispecies plantations across the globe to formulate effective policy level interventions to restore this rapidly degrading and unique ecosystem.

## Figures and Tables

**Table 1:** The location and description of the study sites/metapopulation.

Sites	Name of Monitoring Location	Latitude and Longitudes	Approximate Linear Distance from Nearest Conserved Mangrove Forest Patch—Sajnekhali Sanctuary (km)	Remarks
S1	Plantation site	22.1013272° N, 88.855648° E	0.3	The plantation comprises of *Rhizophora mucronata*, executed in 2012. The plantation site is behind a natural *Avicennia marina* plantation site, as depicted in detail in Figure 2. Hence, the study site is an association between naturally grown A. marina and planted *R. mucronata.*
S2	Lahiripur	22.0912318° N, 88.9027087° E	0.3	Near to the Lahiripur Revenue village. The patch is dominated by *Avicennia marina*.
S3	Poroshmoni sub-center	22.1210751° N, 88.8902425° E	3.7	The area is in a creek locally called ‘Dutta River’. It has a 60% Relative density for *Avicennia marina,* while *Sonneretia caseolaris* constitute another 20%. This is a natural association of *Avicennia*-*Sonneretia*.
S4	Amlamethi Mudflat	22.0807666° N, 88.7105938° E	9	Mudflat in Bali Island of Gosaba block under 80% relative density of *Phoenix paludosa*, the mangrove palm.
S5	Chotto-Mollakhali	22.2281325° N, 88.8487385° E	12	Near to the revenue village of Chotto-Mollakhali. Dominance of halophyte-*Suaeda maritima*

**Table 2:** The biodiversity assessment of the five sites. Relative density (RD) and Frequency (F) occupancy of the species have been represented in the table. As the ‘values’ represented in the table have been ‘approximated’ to nearnest integer, at some sites the total RD value may be above or less than ‘100’.

		Site 1		Site 2		Site 3		Site 4		Site 5	
Species	Family	RD	F	RD	F	RD	F	RD	F	RD	F
*Acanthus ilicifolius* L.	Acanthaceae	8	100	8	100	06	60	10	60	4	60
*Avicennia marina* (Forssk.) Vierh	Acanthaceae	24	60	80	100	55	100	16	100	13	40
*Bruguera sexangula* (Lour.) Poir.	Rhizophoraceae	4	60	1	20	0	0	0	0	0	0
*Ceriops tagal* (Perr.) C.B. Robinson	Rhizophoraceae	1	40	0	0	0	0	0	0	0	0
*Phoenix paludosa* Roxb.	Arecaceae	0	40	0	0	4	60	63	100	4	80
*Proteresia coarctata* (Roxb.) Tateoka	Poaceae	4	0	12	40	13	40	10	20	10	40
*Rhizophora mucronata* Lamk.	Rhizophoraceae	60	100	0	0	0	40	0	0	0	0
*Sonneretia caseolaris* (L.) Engl.	Sonnetiaceae	0	0	0	0	21	100	0	0	0	0
*Suaeda maritima* (L.) Dumort.	Amaranthaceae	0	0	0	0	0	0	0	0	69	100

**Table 3:** Comparison of Simpson’s Index of Dominance/Diversity and Shannon-Weiner Index between the five sites.

	Site 1	Site 2	Site 3	Site 4	Site 5
Simpson’s Index of Dominance	0.41	0.66	0.36	0.43	0.5
Simpson’s Index of Diversity	0.59	0.34	0.64	0.57	0.5
Shannon-Weiner Index	1.13	0.65	1.23	1.05	1.0

**Table 4:** Soil parameters of the sampled sites from S1 to S5, with mean and standard deviation (±). ANOVA were performed, alphabets (a to d) are indicating Duncan’s Multiple Range Tests (DMRT).

	Sand	Silt	Clay	pH	SOC (%)	BD (g/cm^3^)	P (mg/kg)	Salinity (ppt)	N (mg/kg)
S1	6.8 ± 0.5 ^a^	36.2 ± 4.9 ^a^	57.0 ± 5.2 ^d^	7.6 ± 0.1 ^a^	1.9 ± 0.12 ^c^	1.8 ± 0.1 ^b^	9.4 ± 1.2 ^b^	9.3 ± 0.4 ^a^	74.2 ± 3.5 ^b^
S2	10.4 ± 0.8 ^c^	40.4 ± 1.8 ^ab^	49.2 ± 1.6 ^c^	7.4 ± 0.1 ^a^	1.8 ± 0.09 ^c^	1.7 ± 0.05 ^b^	8.5 ± 0.7 ^b^	8.9 ± 0.3 ^a^	72.4 ± 8.2 ^b^
S3	8.7 ± 1.4 ^b^	64.2 ± 3.7 ^c^	27.1 ± 3.6 ^a^	7.8 ± 0.2 ^a^	1.0 ± 0.06 ^b^	1.5 ± 0.1 ^a^	9.0 ± 0.5 ^b^	7.9 ± 0.26 ^a^	83.6 ± 4.5 ^c^
S4	19.3 ± 1.1 ^d^	44.8 ± 2.6 ^b^	35.9 ± 3.5 ^b^	11.3 ± 1.2 ^b^	0.8 ± 0.11 ^a^	1.8 ± 0.08 ^b^	5.0 ± 0.5 ^a^	16.5 ± 1.5 ^b^	45.2 ± 3.3 ^a^
S5	19.0 ± 0.8 ^a^	60.8 ± 2.3 ^a^	20.2 ± 1.6 ^d^	11.7 ± 0.6 ^a^	0.7 ± 0.05 ^c^	1.4 ± 0.1 ^b^	3.0 ± 0.2 ^b^	18.0 ± 2.4 ^a^	37.2 ± 3.5 ^b^
F value	97.8	28.9	32.5	29.8	78.54	8.4	14.7	65.6	25.7

## Data Availability

Data is presented in the manuscript and the raw data is with the authors.
